# Comparison of Different Methods for Assaying the In Vitro Activity of Cefiderocol against Carbapenem-Resistant *Pseudomonas aeruginosa* Strains: Influence of Bacterial Inoculum

**DOI:** 10.3390/antibiotics13070663

**Published:** 2024-07-18

**Authors:** Celia García-Rivera, Antonia Sánchez-Bautista, Mónica Parra-Grande, Andrea Ricart-Silvestre, María Paz Ventero, Iryna Tyshkovska, Esperanza Merino, Juan Carlos Rodríguez Díaz

**Affiliations:** 1 Microbiology Department, Dr. Balmis University General Hospital, Alicante Institute for Health and Biomedical Research (ISABIAL), 03010 Alicante, Spain; parra_mongra@gva.es (M.P.-G.); ricart_and@gva.es (A.R.-S.); ventero_mar@isabial.es (M.P.V.); iryna_t90@hotmail.es (I.T.); rodriguez_juadia@gva.es (J.C.R.D.); 2 Infectious Diseases Unit, Dr. Balmis University General Hospital, Alicante Institute for Health and Biomedical Research (ISABIAL), 03010 Alicante, Spain; merino_luc@gva.es; 3 División de Microbiología, Universidad Miguel Hernández, Apartado 18, 03550 San Juan de Alicante, Spain

**Keywords:** cefiderocol, inoculum, *Pseudomonas aeruginosa*, in vitro activity, antibiogram, resistance

## Abstract

Carbapenem-resistant *Pseudomonas aeruginosa* infections represent a critical public health concern, highlighting the need for the development of effective antibiotics. Cefiderocol demonstrated potent in vitro activity against *Pseudomonas aeruginosa*, particularly in strains that are resistant to other drugs. However, concerns regarding the emergence of drug-resistant strains persist. This study, conducted with 109 carbapenem-resistant *Pseudomonas aeruginosa* strains from the Spanish Hospital (Dr. Balmis, Alicante). The study evaluated susceptibility to cefiderocol in comparison to alternative antibiotics and including their susceptibility to bacterial inoculum, while assessing various testing methods. Our findings revealed high susceptibility to cefiderocol against carbapenem-resistant strains, with only 2 of 109 strains exhibiting resistance. Comparative analysis demonstrated superiority of cefiderocol towards alternative antibiotics. Both the E-test and disk-diffusion methods showed 100% concordance with the microdilution method in classifying strains as susceptible or resistant. However, 4.6% (5/109) of disc zone diameters fell within the technical uncertainty zone, so the E-test technique was found to be more useful in routine clinical practice. Additionally, escalating bacterial inoculum correlated with decreases in vitro activity, so this parameter should be adjusted very carefully in in vivo studies. This study underscores cefiderocol’s potential as a therapeutic option for carbapenem-resistant *Pseudomonas aeruginosa* infections. However, the emergence of drug-resistant strains emphasizes the critical need for a wise use of antibiotics and a continuous monitoring of resistance to antibiotics. Based on our in vitro data, further investigation concerning the impact of bacterial inoculum on drug efficacy is warranted in order to detect resistance mechanisms and optimize treatment strategies, thereby mitigating the risk of resistance.

## 1. Introduction

Severe infections caused by carbapenem-resistant *Pseudomonas aeruginosa*, attributed to the production of metallo-β-lactamases, entail a significant public health challenge. These infections are characterized by high morbidity and mortality rates and a scarcity of available therapeutic alternatives. This pathogen exhibits inherent resistance to many drug types, including aminopenicillins, first- and second-generation cephalosporins, and some third-generation cephalosporins, tetracyclines, trimethoprim/sulfamethoxazole, among others. Additionally, it can form biofilms, which not only complicate treatment but also facilitate the rapid acquisition of resistance to ongoing therapies [1]. Antibiotic resistance in *Pseudomonas aeruginosa* involves multiple mechanisms such as enzyme production, loss of outer membrane proteins, target mutations, and multidrug efflux systems [2,3].

One of the tools in the fight against antibiotic resistance is the development of new antibiotics that target these microorganisms. Consequently, drugs from different families have been introduced to the market. Among these, we highlight the development of cefiderocol, a cephalosporin with potent in vitro activity against *Pseudomonas aeruginosa* strains that are resistant to other β-lactam drugs [4]. It has a peculiar mechanism of action because it is absorbed through iron transport channels by active transport. It also enters bacteria through traditional porin channels. In addition, cefiderocol is relatively stable against the hydrolysis of most serine and metallo-β-lactamases, including the carbapenemases KPC, NDM, VIM, IMP, and OXA [5]. However, the contribution of beta-lactamases, especially certain enzyme variants, in the occurrence of resistance to or reduced effectiveness of cefiderocol has also been demonstrated by in vitro synergy assays with clinically available beta-lactamase inhibitors [6].

This new antibiotic is emerging as a significant therapeutic alternative due to the challenges presented by infections caused by carbapenem-resistant *Pseudomonas aeruginosa* strains producing metallo-β-lactamases, particularly the VIM variant. These infections are difficult to treat and represent a major health concern in our environment due to limited therapeutic options. However, the emergence of drug-resistant strains has been reported despite the infrequent use of the drug [7,8,9,10].

From a microbiological standpoint, studying the in vitro activity of the drug is methodologically complex since its mechanism of entry into bacterial cells, involves interaction with iron in the medium. There is little information on the concordance of different microbiological methods in *Pseudomonas aeruginosa*. This study compares three commonly used methods for analyzing the in vitro activity of this compound: microdilution in broth with iron depletion, the E-test, and disc diffusion. It has been reported that increasing the bacterial inoculum decreases the in vitro activity of the drug [11]. Therefore, in this study, we aim at analyzing the variation in the minimum inhibitory concentration (MIC) of the drug by using inoculum levels lower and higher than those established by EUCAST for this parameter analysis

## 2. Results

### 2.1. Study of Cefiderocol in Relation to Other Antimicrobials

Out of a total of 109 strains, 98.20% were susceptible to cefiderocol, with only 2 strains showing resistance. Among the isolates producing VIM-type carbapenemases, susceptibility decreased to 96.3% (two resistant strains that were susceptible exclusively to colistin), while susceptibility in the group of 55 non-metallo-β-lactamases-producing strains was 100%.

Table 1 shows the percentage of susceptibility to cefiderocol in strains susceptible or resistant to other antibiotics used in therapy against *Pseudomonas aeruginosa*. Notably, the two strains resistant to cefiderocol were only susceptible to colistin.

Table 2 shows MIC50 (mg/L) and MIC90 (mg/L) values for cefiderocol compared to other drugs used in the treatment of *Pseudomonas aeruginosa*. It demonstrates that cefiderocol exhibited the highest in vitro activity, with lower MIC50 (mg/L) and MIC90 (mg/L) values.

### 2.2. Analysis of the Different Techniques for Susceptibility to Cefiderocol

This study compares two cefiderocol susceptibility techniques—the diffusion gradient strips E-test Liofilchem^®^ (Roseto degli Abruzzi, Italy) and Thermo Scientific™ Oxoid™ (Waltham, MA, USA) antimicrobial susceptibility discs—with the reference method, microdilution in iron-depleted medium using UMIC^®^ Bruker (Bruker Daltonics GmbH & Co. KG, Bremen, Germany). For this purpose, the reference inoculum used in the cefiderocol susceptibility study was 1.5 × 10^8^ CFU (colony forming unit)/mL (0.5 McFarland).

**Figure 1:**
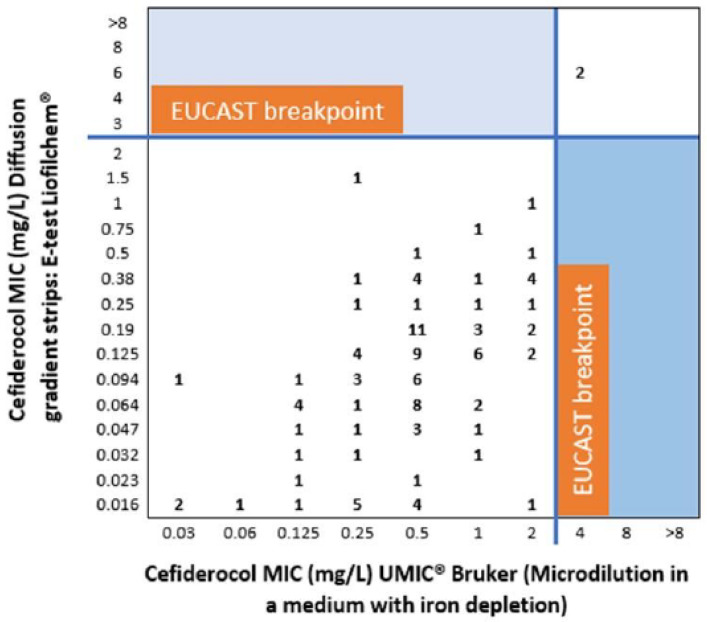
Comparison of microdilution in a medium with iron depletion, UMIC^®^ Bruker with diffusion gradient strips, and E-test Liofilchem^®^.

**Figure 2:**
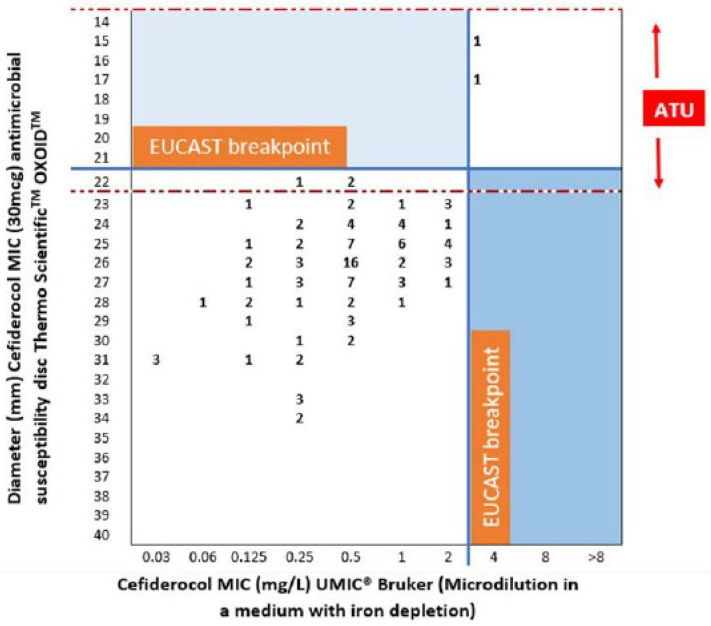
Comparison of microdilution in a medium with iron depletion: UMIC^®^ Bruker with Thermo Scientific™ Oxoid™ cefiderocol antimicrobial susceptibility discs. (ATU: Area of technical uncertainty).

These data are presented in Figure 1 and Figure 2, where the evaluated technique is plotted on the y-axis against the reference technique on the x-axis. The MIC values are also shown (mg/L). When comparing the diffusion gradient strips E-test BD (Liofilchem^®^) method to the reference method (Figure 1), we observed 100% of categorical agreement. Likewise, Thermo Scientific™ Oxoid™ antimicrobial susceptibility disc method (Figure 2) achieved 100% of categorical agreement, but the two strains that are resistant to cefiderocol (MIC 4 mg/L) generated inhibition zone diameters falling within the area of technical uncertainty (ATU).

### 2.3. Investigation of Inoculum Influence

To study the influence of the inoculum, two different techniques were used: diffusion gradient strips, E-test Liofilchem^®^ of cefiderocol and Thermo Scientific™ Oxoid™ cefiderocol antimicrobial susceptibility discs, both in Mueller Hinton E Agar (BioMérieux Espãna S.A.—Manuel Tovar, 45–47, 28 034 Madrid). Three different inoculum concentrations were used. From smallest to largest, these included 1.5 × 10^7^ CFU/mL, 1.5 × 10^8^ CFU/mL and 3 × 10^8^ CFU/mL. Increasing the inoculum concentration in all *Pseudomonas aeruginosa* isolates resulted in higher MIC values and reduced antibiotic in vitro activity, without changes in the susceptible/resistant category in either technique. Additionally, with Thermo Scientific™ Oxoid™ cefiderocol antimicrobial susceptibility discs, it increased the inoculum-generated inhibition zone diameters falling within the area of technical uncertainty (ATU) (15 and 17 mm halo diameters).

A statistical analysis was conducted to study the influence of the inoculum in both evaluated techniques, comparing the three groups of initial inoculum concentrations (1.5 × 10^7^ CFU/mL, 1.5 × 10^8^ CFU/mL, and 3 × 10^8^ CFU/mL). The data showed that the data did not follow a normal distribution. Therefore, the test used to assess the distribution among the different inoculum groups was the Kruskal–Wallis test. This test yielded a result with a *p*-value < 5%, indicating statistically significant differences among the three groups of initial concentrations (CFU/mL) in both techniques. To enhance the analysis, pairwise comparisons were also conducted using the Dunn’s test, which also revealed significant differences in the MIC values based on the initial inoculum used.

## 3. Discussion

The use of mass spectrometry (MALDI TOF) for the identification of pathogenic microorganisms has made it possible to obtain information on the etiology of the process before studying the in vitro activity of the drugs. In addition to rapid tests for the detection of resistance mechanisms, information on the epidemiology of antibiotic resistance in each environment has become increasingly important as it helps us to position new drugs within the available therapeutic arsenal, especially in situations where empirical treatments need to be established or antibiotic treatments need to be rapidly adjusted. In addition to the benefit to the individual patient, the stewardship groups the benefits from this information to design policies for the prevention and control of serious infections in each center, as it allows them to determine the evolution of the frequency of resistant strains in each setting [12,13,14].

Our study was conducted on clinical isolates prior to drug usage, establishing a baseline for monitoring resistance evolution. On one hand, this process is now particularly important due to the increasing use of the drug in clinical practice. It has been reported that antibiotic resistance usually develops in the months following increased antibiotic use [15,16]. On the other hand, although there are strains that are resistant to cefiderocol which are not producers of carbapenemsases and which therefore have other mechanisms of resistance, we have not detected the presence of these strains in our environment [17].

In our clinical isolates from patients diagnosed before the drug’s introduction, we detected only two strains that were resistant to the drug. These were associated with carbapenem resistance due to production of the carbapenemase VIM. These data coincide with previously published results [18,19]. Repeatedly confirmed findings indicate that the drug’s activity is strongly influenced by the type of carbapenemases. It exhibits high activity against the VIM type, but its effectiveness decreases against New Delhi metallo-β-lactamases (NDM) (83.4%) [20]. Therefore, when it comes to the clinical management of this drug, it is very important to conduct a rapid test that detects the absence of carbapenemases and/or the type of carbapenemase present. The low antibiotic resistance to the drug poses a methodological limitation due to the low number of resistant strains. However, this reflects real-life conditions at present [21]. Similarly, there are no data available in our environment regarding the drug activity on strains carrying other types of carbapenemases.

Karakonstantis S et al. performed a meta-analysis and reported that cefiderocol showed activity against most carbapenem-resistant *Pseudomonas aeruginosa* clinical isolates, including in metallo-β-lactamases producers, although it is recommended that one should monitor the evolution of resistance to this compound. Their analysis of 82,035 clinical isolates of Gram-negative bacilli revealed a very low percentage of resistant strains (1.4% [95% CI 0.5–4.0%]), reaching a conclusion that coincides with our findings [20].

Our data, obtained from the studied strains, show that cefiderocol is an important therapeutic tool for the treatment of difficult-to-treat *Pseudomonas aeruginosa*-associated infections compared to the other antibiotics studied, due to its good in vitro activity [22,23,24,25].

Our study utilized three microbiological methods to compare their efficacy. Our findings highlight the E-test as a viable alternative in laboratories lacking specific iron-depleted media, as its results aligned with those obtained from the micro-dilution system with iron depletion, recognized as the gold standard [26]. In contrast, the disk-diffusion method placed two resistant strains in a zone of technical uncertainty, casting doubt on its clinical utility. Our data shed light on this contentious issue. Devoos et al. suggested limited utility of the E-test and reported that disks often fail to detect resistant strains, while Matuschek et al. argued that disk diffusion is robust [27,28]. Bianco et al. analyzed various species, particularly enterobacteria, and found good correlation between the disk diffusion and microdilution systems, though many strains fell into the technically uncertain category, which is with our findings [29]. Furthermore, a novel system analyzing microbial glucose metabolism in the presence of antibiotics shows promising results as an alternative to these traditional methods [30].

Regarding the influence of the initial bacterial inoculum used, we observed a decrease in the intrinsic activity of the drug as the starting inoculum was increased. Therefore, an inoculum quality control system must be included in this process. Despite this fact, the strains still fell into either the susceptible or resistant category, which contradicts previously published data indicating that, when facing the use of high inoculum, a change in category from susceptible to resistant was observed in most of the isolates of carbapenem-resistant Enterobacterales [31,32]. These data were previously in agreement, indicating that the inoculum’s effect has little influence on the activity of carbapenems against this pathogen in animal models [33].

According to our in vitro results, this decrease in intrinsic drug activity at a high level of bacterial inoculum could be one of the causes explaining the risk of the appearance of treatment-resistant mutants in high-inoculum infections, which is beginning to be reported despite the low use of the drug [32]. Thus, a small proportion of resistant strains associated with various mechanisms generated by bacteria during treatment have been reported: mutations affecting porins, siderophore receptors, and efflux pumps, as well as modifications to the target (PBP-3) [34,35]. Another phenomenon to highlight is the emergence of persistence and hypermutation due to antibiotic treatment failure [36,37]. Furthermore, the contribution of beta-lactamases, especially certain enzyme variants, in the occurrence of resistance or reduced sensitivity to cefiderocol has been also demonstrated by in vitro synergy assays with clinically available beta-lactamase inhibitors [6].

The clinical significance and causes of the inoculum’s effect on this drug are not well understood, but biofilms may be involved in high-inoculum infections, such as respiratory infections (inoculum of 10^8^ UFC/mL). Additionally, quorum sensing has been reported to produce proteins that decrease antibiotic susceptibility. Moreover, the presence of a large bacterial inoculum promotes the survival of minority subpopulations with preexisting resistance mechanisms. There is little clinical evidence of the importance of the inoculum’s effect, although the decreased efficacy of drugs has been demonstrated in animal models. Differences between drugs have been demonstrated; thus, the analysis of this parameter in this new drug with its own mechanism of entry into the bacteria provides relevant information that needs to be confirmed by further studies.

Despite the limited use of the drug, the emergence of resistant strains is concerning. A meta-analysis reported that 1.4% of *Pseudomonas aeruginosa* strains are resistant to cefiderocol [21]. Cross-resistance with ceftolozane-tazobactam has also been reported [38]. Therefore, microbiologists must closely monitor the evolution of antibiotic resistance in each setting to control this phenomenon.

Proper utilization of this drug and prevention of resistant mutant emergence necessitates promoting 24/7 microbiological diagnostics to ensure continuous detection of phenotypic susceptibility using the E-test and continuous surveillance of the emergence of resistant strains to limit their spread to other patients. The presence of carbapenemases and the type of carbapenemase is a key factor in determining the in vitro activity of the drug. Additionally, implementing a strict antibiotic stewardship policy is essential to ensure appropriate usage of this drug, specifically when warranted and under optimal conditions, allowing it to remain one of the best therapeutic alternatives for carbapenem-resistant *Pseudomonas aeruginosa* infections, especially in infections with a high inoculum, to combat the risk of generation of resistant mutants during treatment [39].

## 4. Materials and Methods

The study was carried out using 109 strains of carbapenem-resistant *Pseudomonas aeruginosa* obtained consecutively from samples received at the clinical microbiology laboratory of Dr. Balmis Hospital (Alicante) between 2021 and 2022, prior to the use of this antibiotic. Pathogen identification was performed using mass spectrometry (MALDI-TOF) from the pathogen culture. The obtained strains were stored at −80 °C in 1.5 mL Eppendorf tubes containing a prepared solution of Trypticase Soy Broth (TSB-T) and glycerol. For the use of the pathogen in the study, the tubes containing the microorganism were thawed at room temperature. Once thawed, a transfer was made from the liquid medium to blood agar, and it was incubated for 24 h at 37 °C to finally obtain the isolate for processing. Of the 109 strains of *Pseudomonas aeruginosa*, there were 54 consecutive isolates producing VIM-type carbapenemases and 55 consecutive isolates without the presence of carbapenemases; the latter were resistant to carbapenem through other mechanisms.

The strains were obtained consecutively from the Hospital General Dr. Balmis (Alicante, Spain) and were a representative sample of the *Pseudomonas aeruginosa* found in our setting.

Antibiotic susceptibility: The determination of antibiotics susceptibility, including carbapenems, was performed using the Walk Away system (Beckman Coulter); in addition, since the study was conducted with clinical strains that showed resistance to carbapenems, an additional test was performed to detect carbapenemase production using Cepheid GeneXpert^®^ Systems and immunochromatography NG-Test CARBA 5, Biotech (Z.A. Courbouton, secteur 1 Atelier relais le Tremplin 35480 Guipry, France.

In the 109 strains, cefiderocol susceptibility was compared with those towards imipenem/relebactam, ceftolozane/tazobactam, colistin, amikacin, piperacillin/tazobactam, ceftazidime, and cefepime. Antimicrobials susceptibility aside from cefiderocol was tested by using gradient strips E-test Liofilchem^®^.

The in vitro activity of cefiderocol was studied using three different methods:

- Microdilution in iron-depleted medium (UMIC^®^ Bruker): The UMIC^®^ Cefiderocol is suitable for determining MIC values in the range of 0.03 to 32 mg/L for *Pseudomonas aeruginosa*. UMIC are ready-to-use strips with 12 wells each, containing dried antibiotics and prepared according to the ISO 20776-1:2019 [40]. First, a 0.5 McFarland (1.5 × 10^8^ CFU/mL) standard bacterial suspension is prepared in sodium chloride 0.9% solution; then, it is transferred to a 25 μL aliquot of cation-adjusted, iron-depleted Mueller Hinton broth. Next, UMIC cefiderocol was inoculated by transferring 100 μL to each well with the appropriate number of UMIC strips. The UMIC BOX was incubated for 18–24 h until we were able to visually read the MIC results.
- Diffusion gradient strips E-test Liofilchem^®^ of cefiderocol: This technique was carried out according to the manufacturer’s instructions using three different bacterial inoculums. The reference or standard inoculum corresponded to 1.5 × 10^8^ CFU/mL (0.5 McFarland), which is a lower inoculum than the standard which corresponded to 1.5 × 10^7^ CFU/mL; finally, a higher inoculum than the standard corresponded to 3 × 10^8^ CFU/mL (1 McFarland). All of them used plates with Mueller Hinton E Agar (Biomerieux).
- Thermo Scientific™ Oxoid™ cefiderocol antimicrobial susceptibility discs: This technique was carried out according to the manufacturer’s instructions using three different bacterial inoculums. The reference or standard inoculum corresponded to 1.5 × 10^8^ CFU/mL (0.5 McFarland); a lower inoculum than standard corresponded to 1.5 × 10^7^ CFU/mL; finally, a higher inoculum than standard corresponded to 3 × 10^8^ CFU/mL (1 McFarland). All of them used plates with Mueller Hinton E Agar (Biomerieux).

Statistical analysis was performed to determine whether statistically significant differences could be found between the three initial inoculums (1.5 × 10^8^ CFU/mL, 1.5 × 10^7^ CFU/mL and 3 × 10^8^ CFU/mL) using diffusion gradient strips of E-test Liofilchem^®^. To check whether the data followed a normal distribution, we applied the Shapiro–Wilk test. To study whether there were statistically relevant differences between the three groups of data, the Kruskal–Wallis test was used; finally, to compare groups 2 to 2, Dunn’s test with the Benjamini–Hochberg correction was used.

Interpretation of the results was performed using the EUCAST 2023 criteria [41].

## Figures and Tables

**Table 1:** In vitro activity of cefiderocol in relation to the activity of the other drugs studied. S = susceptible; R = resistant.

Comparison of % Susceptibility to Cefiderocol vs. Other Antibiotics	
Other Antibiotics	S Cefiderocol
S: Imipenem/relebactam (*n* = 9)	100%
R: Imipenem/relebactam (*n* = 100)	98%
S: Ceftolozane/tazobactam (*n* = 56)	100%
R: Ceftolozane/tazobactam (*n* = 53)	96.20%
S: Colistin (*n* = 108)	98.20%
R: Colistin (*n* = 1)	100%
S: Amikacin (*n* = 57)	100%
R: Amikacin (*n* = 52)	96.15%
S: Piperacillin/Tazobactam (*n* = 27)	100%
R: Piperacillin/Tazobactam (*n* = 82)	97.50%
S: Ceftazidime (*n* = 38)	100%
R: Ceftazidime (*n* = 71)	97.18%
S: Cefepime (*n* = 42)	100%
R: Cefepime (*n* = 67)	97.02%
TOTAL (*n* = 109)	98.20%
Metallo-β-lactamases producers (*n* = 54)	96.30%
Non- metallo-β-lactamases producers (*n* = 55)	100%

**Table 2:** Results of MIC50 (mg/L) and MIC90 (mg/L) values for cefiderocol compared to other drugs.

ANTIBIOTICS	MIC_50_	MIC_90_
Cefiderocol	0.11	0.38
Imipenem/relebactam	>64	>64
Ceftolozane/tazobactam	3	>256
Colistin	0.125	4
Amikacin	16	>256
Piperacillin/tazobactam	48	64
Ceftazidime	16	>32
Cefepime	16	>16

## Data Availability

The original contributions presented in the study are included in the article, further inquiries can be directed to the corresponding authors.
